# Ecocardiograma Transtorácico pode Substituir Radiografia de Tórax na Avaliação de Cardiomegalia na Miocardiopatia Chagásica?

**DOI:** 10.36660/abc.20200625

**Published:** 2021-01-27

**Authors:** Tiago Senra

**Affiliations:** 1 Instituto Dante Pazzanese de Cardiologia São PauloSP Brasil Instituto Dante Pazzanese de Cardiologia, São Paulo, SP – Brasil

**Keywords:** Miocardiopatia Chagásica, Insuficiência Cardíaca, Cardiomegalia, Morbimortalidade, Epidemiologia, Aspectos Socioeconômicos, Ecocardiografia/métodos, Radiografia Torácica/métodos

A miocardiopatia chagásica (MC), descrita originalmente em 1909 por Carlos Chagas,^1^ segue nos dias atuais associada a elevada morbimortalidade e impacto socioeconômico, especialmente nos países da America Latina.

Nesse sentido, a busca por marcadores epidemiológicos, clínicos, laboratoriais, eletrocardiográficos e de imagem associados ao prognóstico cumpre papel importante na estratificação de risco desses pacientes.

O índice cardiotorácico (ICT), originalmente descrito em 1919 e calculado a partir da radiografia de tórax é uma das variáveis associadas à mortalidade na MC.

Rodriguez-Salas et al.,^2^ identificaram o ICT>0,55 como variável independente associada à mortalidade em estudo que incluiu 960 pacientes. Notadamente, o diâmetro diastólico final do ventrículo esquerdo (DDVE) avaliado pelo ecocardiograma transtorácico não se associou à má evolução nesse estudo.

Posteriormente, Salles et al.,^3^ detectaram associação entre o ICT>0,5 e mortalidade por todas as causas em estudo que incluiu 738 pacientes, mas o diâmetro sistólico final do ventrículo esquerdo medido pelo ecocardiograma transtorácico foi variável independente mais fortemente associada à morte por todas as causas, além de morte relacionada à MC e morte súbita. Esse achado possivelmente reflete melhor a relação entre disfunção sistólica ventricular esquerda e má evolução que o aspecto morfológico dessa câmara.

Por outro lado, Bestetti et al.,^4^ identificaram o DDVE analisado pelo ecocardiograma transtorácico como preditor independente de morte súbita em estudo que incluiu 74 pacientes e não mostrou associação entre ICT alterado e o desfecho.

Finalmente, Rassi et al.,^5^ conduziram um estudo retrospectivo incluindo 424 pacientes que identificou o ICT>0,5 como variável independente associada à mortalidade, com razão de risco de 3,43, superior à razão de risco relacionada à alteração global ou segmentar da contratilidade do ventrículo esquerdo identificada pelo ecocardiograma transtorácico de 2,46. Esses achados contribuíram para a elaboração do escore de Rassi, que atribui mais pontos^5^ para a cardiomegalia identificada na radiografia de tórax que a disfunção ventricular esquerda avaliada pelo ecocardiograma^3^ e foi validado em coorte independente. Notadamente, apesar de ter sido incluído como variável no estudo, o DDVE não mostrou associação com o desfecho e não foi incluído no escore, que conta ainda com classe funcional de insuficiência cardíaca, taquicardia ventricular não-sustentada, baixa voltagem e sexo masculino.

Nesse contexto, autor Ramos et al.^6^ avaliaram a correlação entre o ICT e o DDVE num grupo de 58 pacientes com MC e apresentam seus resultados na atual edição dos Arquivos Brasileiros de Cardiologia.

Os autores encontraram boa acurácia (área sob a curva ROC 0,806) à custa principalmente de especificidade elevada (89%) e sensibilidade moderada (64%) para um limiar de DDVE de 60mm na detecção de cardiomegalia identificada pela radiografia de tórax.

A substituição do ICT pelo DDVE para definição do escore de Rassi resultou na reclassificação com mudança de patamar em 24% dos pacientes, a maioria com redução da faixa de risco.

Ainda que a radiografia de tórax acarrete exposição do paciente à radiação, o que não ocorre com o ecocardiograma, o argumento de que a substituição do método radiológico pelo ultrassonográfico possa ser benéfica ao paciente não parece justificado, visto que a dose de radiação de uma radiografia de tórax é mínima e seu eventual potencial carcinogênico na população incluída no estudo (média de 61 anos) seja questionável.

Por outro lado, é inegável que todo paciente com sorologias positivas para Chagas e sinais/sintomas de acometimento cardíaco deve realizar ecocardiograma como parte de sua investigação e estratificação de risco. Uma ótima correlação entre ICT e DDVE poderia dispensar a radiografia de tórax, com vantagem prática na rotina, mas muito provavelmente sem impacto significativo no custo.

A sensibilidade de 64% do DDVE para diagnóstico de cardiomegalia na comparação com a radiografia de tórax detectada no estudo preocupa, especialmente se lembrarmos que essa variável assume tamanha importância no escore de Rassi, superior à disfunção ventricular esquerda global/segmentar, podendo levar à reclassificação errônea para faixas menores de risco.

A melhor forma de confirmar se os novos valores do escore de Rassi determinados pela substituição do ICT pelo DDVE melhora a estratificação de risco será o seguimento da coorte, já proposto pelos autores.

Análises adicionais como a regressão linear entre os valores de ICT e DDVE, análise de Bland-Altmann, de concordância kappa e a indexação do DDVE pela superfície corpórea podem fornecer dados interessantes para elucidar as discrepâncias entre os dois métodos.

Nessa linha, o acometimento segmentar na MC pode estar subdimensionado ao avaliarmos a morfologia de uma estrutura tridimensional e complexa como ventrículo através de uma medida simplificada como o DDVE^7^ ao invés do volume diastólico final. De fato, estudos prévios^8 , 9^ identificaram o volume diastólico final aumentado como variável associada à mortalidade na MC.

Mesmo com suas próprias limitações, é possível que o ICT represente melhor o acometimento global cardíaco pela patologia do que a avaliação isolada do ventrículo esquerdo por uma medida bidimensional. Na MC, sabemos que o ventrículo direito pode estar acometido de maneira independente do ventrículo esquerdo^10^ e que a disfunção sistólica ventricular direita também está associada ao prognóstico.^11^ Assim, agregar a análise do ventrículo direito poderia melhorar o desempenho do ecocardiograma transtorácico na identificação da cardiomegalia na MC e sua associação com o prognóstico.

Se por um lado a radiografia de tórax ainda parece manter seu lugar na avaliação de pacientes com MC, novas pesquisas em ecocardiografia são promissoras.

## References

[1] . Chagas C. Nova tripanozomiaze humana: estudos sobre a morfolojia e o ciclo evolutivo do Schizotrypanum cruzi n. gen., n. sp., ajente etiolojico de nova entidade morbida do homem. Memorias do Instituto Oswaldo Cruz. 1909;1(2):159-218.

[2] . Rodriguez-Salas LA, Klein E, Acquatella H, Catalioti F, Davalos VV, Gomez-Mancebo JR, et al. Echocardiographic and Clinical Predictors of Mortality in Chronic Chagas’ Disease. Echocardiography. 1998;15(3):271-8. 10.1111/j.1540-8175.1998.tb00607.x 11175040

[3] . Salles G, Xavier S, Sousa A, Hasslocher-Moreno A, Cardoso C. Prognostic value of QT interval parameters for mortality risk stratification in Chagas’ disease: results of a long-term follow-up study. Circulation. 2003;108(3):305-12. 10.1161/01.CIR.0000079174.13444.9C 12835217

[4] . Bestetti RB, Dalbo CM, Arruda CA, Correia Filho D, Freitas OC. Predictors of sudden cardiac death for patients with Chagas’ disease: a hospital-derived cohort study. Cardiology. 1996;87(6):481-7. 10.1159/000177142 8904674

[5] . Rassi A, Jr., Rassi A, Little WC, Xavier SS, Rassi SG, Rassi AG, et al. Development and validation of a risk score for predicting death in Chagas’ heart disease. N Engl J Med. 2006;355(8):799-808. 10.1056/NEJMoa053241 16928995

[6] . Ramos MRF, Moreira HT, Volpe GJ, Romano M, Maciel BC, Schmidt A, et al. Correlação entre Cardiomegalia pela Radiografia de Tórax e Diâmetro do Ventrículo Esquerdo pela Ecocardiografia em Pacientes com Doença de Chagas. Arq Bras Cardiol. 2021; 116(1):68-74. 10.36660/abc.20190673 PMC8159503 33566967

[7] . Teichholz LE, Kreulen T, Herman MV, Gorlin R. Problems in echocardiographic volume determinations: echocardiographic-angiographic correlations in the presence of absence of asynergy. Am J Cardiol. 1976;37(1):7-11. 10.1016/0002-9149(76)90491-4 1244736

[8] . Espinosa RA, Pericchi LR, Carrasco HA, Escalante A, Martinez O, Gonzalez R. Prognostic indicators of chronic chagasic cardiopathy. Int J Cardiol. 1991;30(2):195-202. 10.1016/0167-5273(91)90095-7 2010242

[9] . Carrasco HA, Parada H, Guerrero L, Duque M, Duran D, Molina C. Prognostic implications of clinical, electrocardiographic and hemodynamic findings in chronic Chagas’ disease. Int J Cardiol. 1994;43(1):27-38. 10.1016/0167-5273(94)90087-6 8175216

[10] . Moreira HT, Volpe GJ, Marin-Neto JA, Ambale-Venkatesh B, Nwabuo CC, Trad HS, et al. Evaluation of Right Ventricular Systolic Function in Chagas Disease Using Cardiac Magnetic Resonance Imaging. Circ Cardiovasc Imaging. 2017;10(3):e005571. 10.1161/CIRCIMAGING.116.005571 28289020

[11] . Nunes Mdo C, Rocha MO, Ribeiro AL, Colosimo EA, Rezende RA, Carmo GA, et al. Right ventricular dysfunction is an independent predictor of survival in patients with dilated chronic Chagas’ cardiomyopathy. Int J Cardiol. 2008;127(3):372-9. 10.1016/j.ijcard.2007.06.012 17689706

