# Supplementary material for: Ecocardiograma Transtorácico pode Substituir Radiografia de Tórax na Avaliação de Cardiomegalia na Miocardiopatia Chagásica?
Source: Arq Bras Cardiol. 2021 Jan 27;116(1):75–6. [Article in Portuguese] doi: 10.36660/abc.20200625 (PMC8159511; doi:10.36660/abc.20200625)
Supplement: Translation

# Translation: Can Transthoracic Echocardiography Replace Chest Radiography in the Evaluation of Cardiomegaly in Chagas Cardiomyopathy?

**Authors:** Tiago Senra

**Affiliations:** 1 Brazil Instituto Dante Pazzanese de Cardiologia, São Paulo, SP – Brazil

**Keywords:** Chagas, Cardiomyopathy, Heart Failure, Cardiomegaly, Morbimortality, Epidemiology, Socioeconomic Factors, Echocardiography/methods, Radiography, Thoracic/methods

Chagas cardiomyopathy (CCM), first described in 1909 by Carlos Chagas,^1^is still associated with high morbidity and mortality and socioeconomic impact, especially in Latin American countries.

The search for epidemiological, clinical, laboratory, electrocardiographic, and image markers associated with its prognosis plays an important role in the risk stratification of these patients.

The cardiothoracic ratio (CR), originally described in 1919 and calculated from chest radiography imaging, is one of the variables associated with mortality in CCM.

Rodriguez-Salas et al.^2^identified CR>0.55 as an independent variable associated with mortality in a study with 960 patients. Notably, the left ventricular end diastolic diameter (LVDD) assessed by transthoracic echocardiogram was not associated with poor prognosis in this study.

Afterwards, Salles et al.^3^detected an association between CR>0.5 and all-cause mortality in a study including 738 patients, but the left ventricular end-systolic diameter measured by transthoracic echocardiogram was an independent variable most strongly associated with all-cause mortality, in addition CCM mortality and sudden death. This finding could be a more accurate reflection of the relationship between the left ventricular systolic dysfunction and poor evolution compared to the morphological aspect of this chamber.

On the other hand, Bestetti et al.^4^identified the LVDD upon transthoracic echocardiogram as an independent predictor of sudden death in a study with 74 patients and showed no association between altered CR and the outcome.

Finally, Rassi et al.^5^conducted a retrospective study with 424 patients and reported CR>0.5 as an independent variable associated with mortality, with a risk ratio of 3.43, higher than the risk ratio related to global or segmental change in left ventricular contractility of 2.46 identified by transthoracic echocardiogram. These findings contributed to the elaboration of the Rassi score, which attributes more points^5^to the cardiomegaly identified by chest radiography than the left ventricular dysfunction assessed by echocardiogram^3^and was validated in an independent cohort. Notably, despite being included as a variable in the study, the LVDD was not associated with the outcome and was not included in the score, which also considers the functional class of heart failure, non-sustained ventricular tachycardia (NSVT), low voltage and male gender.

In the same context, author Ramos et al.^6^evaluated the correlation between CR and LVDD in a group of 58 patients with CCM and their results are presented in the current edition of Arquivos Brasileiros de Cardiologia.

The authors found good accuracy (area under the ROC curve of 0.806) at the expense of high specificity (89%) and moderate sensitivity (64%) for a 60mm LVDD threshold in the detection of cardiomegaly identified by chest radiography.

The replacement of CR by LVDD to define the Rassi score resulted in a reclassification with a change in level in 24% of the patients, most of them with a reduction in risk range.

Although chest radiography exposes the patient to radiation, unlike the echocardiogram, the argument that the replacement of radiography with ultrasound may be beneficial to the patient does not seem justified, since the radiation dose in a radiography of thorax is minimal and its eventual carcinogenic potential in the population included in the study (average of 61 years) is questionable.

It is undeniable that every patient testing positive for Chagas disease and presenting signs/symptoms of cardiac involvement should undergo echocardiography as part of the investigation and risk stratification. A great correlation between CR and LVDD could discard chest radiography, with a practical advantage in routine but most likely without significant impact on cost.

The sensitivity of 64% of the LVDD for diagnosis of cardiomegaly in comparison with the chest radiography detected in the study is concerning, especially if we bear in mind that this variable assumes an importance in the Rassi score that is superior to the global/segmental left ventricular dysfunction, which may lead to a wrong reclassification in lower risk ranges.

The best way to confirm whether the new values of the Rassi score determined by replacing CR with LVDD improves risk stratification will be the follow-up of the cohort, already proposed by the authors.

Additional analyses such as linear regression between CR and LVDD values, Bland-Altmann analysis, kappa agreement coefficient and indexing of LVDD by the body surface can provide interesting data to elucidate the discrepancies between the methods.

In the same scenario, the segmental involvement in CCM may be undersized upon evaluation of the morphology of a three-dimensional and complex structure like a ventricle by means of a simplified measurement such as the LVDD^7^instead of the end-diastolic volume. In fact, previous studies^8 , 9^identified the increased end-diastolic volume as a variable associated with mortality in CCM.

Even with its own limitations, the CR could possibly better represent the global cardiac involvement by this pathology compared to an isolated assessment of the left ventricle by a two-dimensional method. In CCM, we know that the right ventricle can be affected regardless of the left ventricle^10^and that the right ventricular systolic dysfunction is also associated with prognosis.^11^Thus, gathering the analysis of the right ventricle could improve the accuracy of the transthoracic echocardiogram when it comes to the identification of cardiomegaly in CCM and its association with the prognosis.

If, on one hand, chest radiography still seems to keep its place in the assessment of patients with CCM, further research in echocardiography is promising.
